# Supplementary material for: The Landscape of SNCA Transcripts Across Synucleinopathies: New Insights From Long Reads Sequencing Analysis
Source: Front Genet. 2019 Jul 9;10:584. doi: 10.3389/fgene.2019.00584 (PMC6629766; doi:10.3389/fgene.2019.00584)
Supplement: Supplementary file 1 [file Data_Sheet_1.docx]

**Supplementary Tables & Figures**

**Supplementary Figure 1. Targeted gDNA capture coverage and phasing for the SNCA region.** For each condition, the sequencing coverage, called SNPs, phased blocks, and capture probes are shown. DLB-4 and N-4 have very few phased blocks due to low heterozygosity. (a) Parkinsons samples (b) Normal samples (c) Dementia with Lewy Body samples.

**(a)**

**
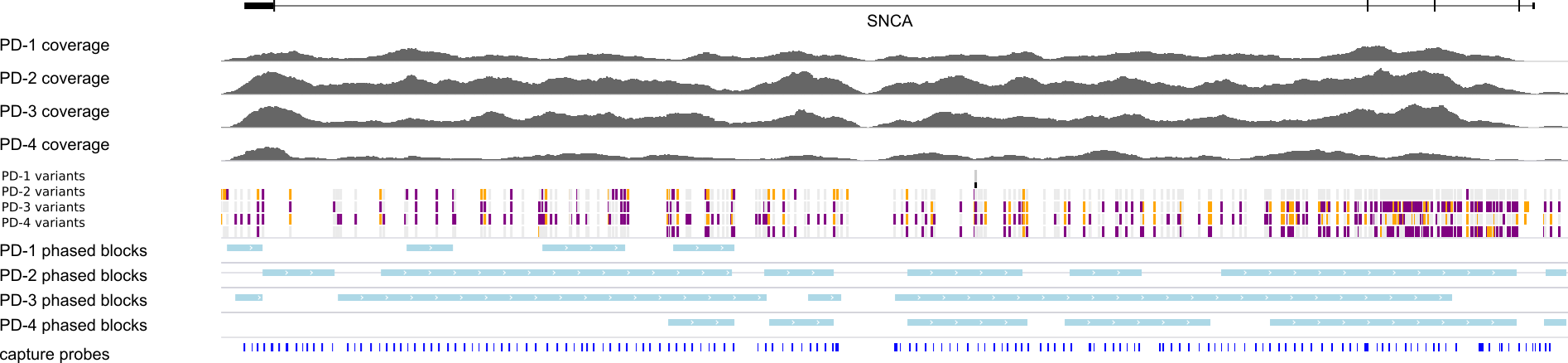
**

**(b)**

**
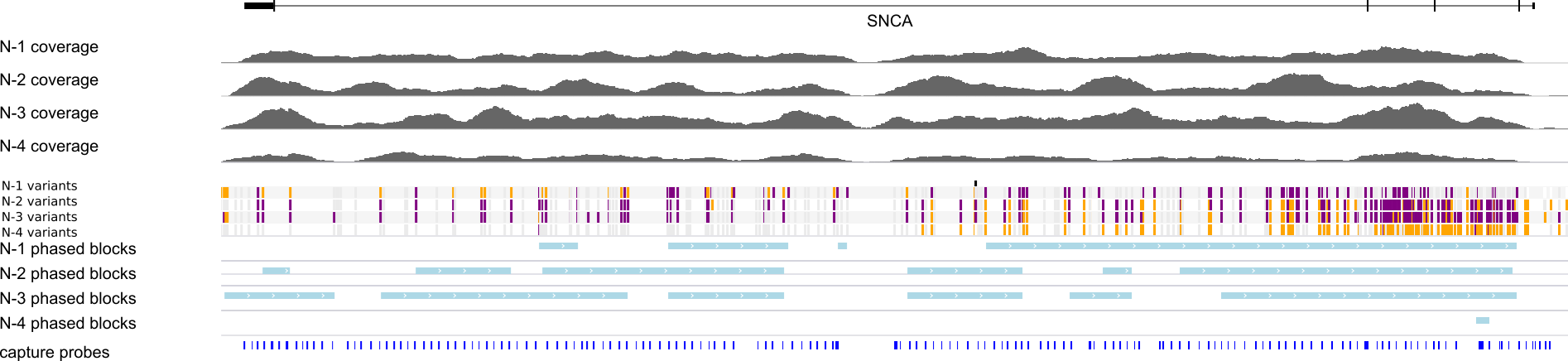
**

**(c)
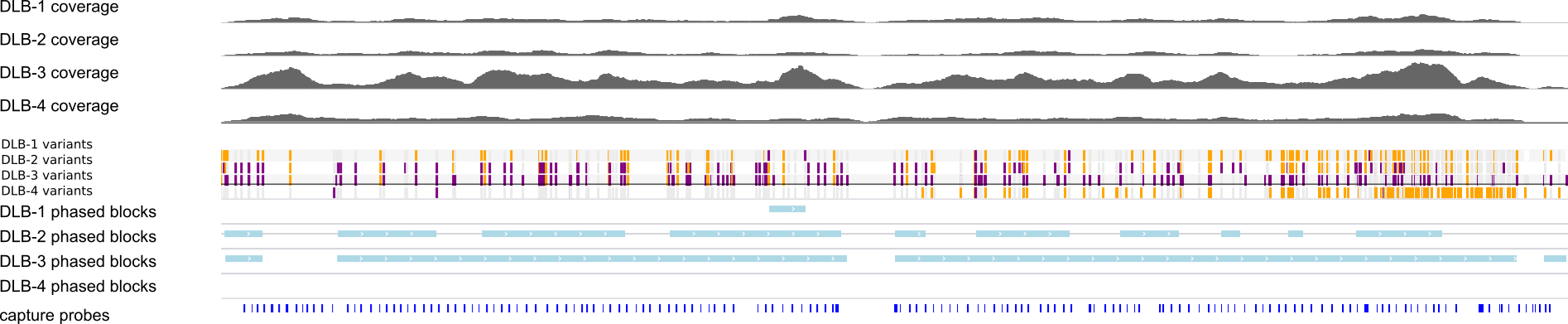
**

**Supplemetary Figure 2. Observed CT-rich region haplotypes for chr4:90742331-90742559 (hs37d5), a previously known repeat in SNCA intron 4.** (a) Haplotypes were determined by extracting, clustering, and calling consensuses independently for each sample. Identical sequences are highlighted. Haplotype 3* differs from Haplotype 3 in a homopolymer region outside of the previous described region.

Haplotype 1 (228bp)

AGTCTCCCTACTTTTTTTCCTTCTTTCTTTCTTTTTCTTTTTCTTTCTTTCTTTCCTTCTTTCTCTCTTTCCTTTCTTTCTTTTCCCTTCCTTCCTTCCTTTCTCCCTTCCTTCCTTCCTCCCTCTCTCCCTCCCTTCCTTCCTCCCTTTCTTTCTTTCTCTTTTTTCTTTCTTGCTTCCTTCCTTCCTTCTTTCCTTTTCTTTCTTTTTCCTTTCTTTGCCAAAGTG

Haplotype 2 (226bp)

AGTCTCCCTACTTTTTTTCCTTCTTTCTTTCTTTTTCTTTTTCTTTCTTTCTTTCCTTCTTTCTCTCTTTCCTTTCTTTCTTTACTTTCTTTCCTTTCTTTCTTTTCCCTTCCTTCCTCCCTCTCTCCCTCCCTTCCTTCCTCCCTTTCTTTCTTTCTCTTTTTTCTTTCTTGCTTCCTTCCTTCCTTCTTTCCTTTTCTTTCTTTTTCCTTTCTTTGCCAAAGTG

Haplotype 3 (250bp)

AGTCTCCCTACTTTTTTTCCTTCTTTCTTTTTTTTTCTTTTTCTTTCTTTCTTTCCTTCTTTCTCTCTTTTCTTTCTTTCTTTACTTTCTTTCCTTTCTTTCTTTTCCCTTCCTTCCTTCCTTTCTCCCTTCCTTCCTTCCTCCCTCTCTCCCTCCCTTCCTTCCTCCCTTTCTTTCTTTCTCTTTTTTCTTTCTTGCTTCCTTCCTTCCTTCTTTCCTTTTCTTTCTTTTTCCTTTCTTTGCCAAAGTG

Haplotype 3* (249bp)

AGTCTCCCTACTTTTTTTCCTTCTTTCTTTCTTTTTCTTTTTCTTTCTTTCTTTCCTTCTTTCTCTCTTTTCTTTCTTTCTTTACTTTCTTTCCTTTCTTTCTTTTCCCTTCCTTCCTTCCTTTCTCCCTTCCTTCCTTCCTCCCTCTCTCCCTCCCTTCCTTCCTCCCTTTCTTTCTTTCTCTTTTT‑CTTTCTTGCTTCCTTCCTTCCTTCTTTCCTTTTCTTTCTTTTTCCTTTCTTTGCCAAAGTG

Haplotype 4 (327bp)

AGTCTCCCTACTTTTTTTCCTTCTTTCTTTCTTTTTCTTTTTCTTTCTTTCTTTCCTTCTTTCTCTCTTTTCTTTCTTTCTTTACTTTCTTTCCTTTCTTTCTTTTCCCTTCCTTCCTTCCTTCTTCCCTTCCTTCCTTTCTCCCTTTCTTTCTTTCTCTTTTTTCTTTCTTGCTTCCTTCCTTCCTTCTTTCCTTTTCTTTCTTTTCCCTTCCTTCCTCCCTCTCTCCCTCCCTTCCTTCCTCCCTTTCTTTCTTTCTCTTTTTTCTTTCTTGCTTCCTTCCTTCCTTCTTTCCTTTTCTTTCTTTTTCCTTTCTTTGCCAAAGTG

(b) Sample genotypes for **CT-rich region as shown in Supp Figure 2a.**

| **Sample** | **Genotype** |
| --- | --- |
| **PD-1** | Hap1/Hap1 |
| **PD-2** | Hap2/Hap4 |
| **PD-3** | Hap2/Hap3 |
| **PD-4** | Hap1/Hap4 |
| **N-1** | Hap1/Hap2 |
| **N-2** | Hap2/Hap4 |
| **N-3** | Hap2/Hap4 |
| **N-4** | Hap4/Hap4 |
| **DLB-1** | Hap2/Hap2 |
| **DLB-2** | Hap2/Hap3* |
| **DLB-3** | Hap1/Hap3 |
| **DLB-4** | Hap4/Hap4 |

**Supplementary Figure 3**. **Phased block size and variant count distribution using targeted gDNA data.** (a) Each point in each sample represents a phased block and the Y-axis indicates the phased block size. Some of the samples (ex: PD-1, N-4, DLB-1) have fewer and shorter block sizes due to low heterozygosity. (b) Each point in each sample represents a phased block and the Y-axis indicates the number of variants in that block.

**(a)**


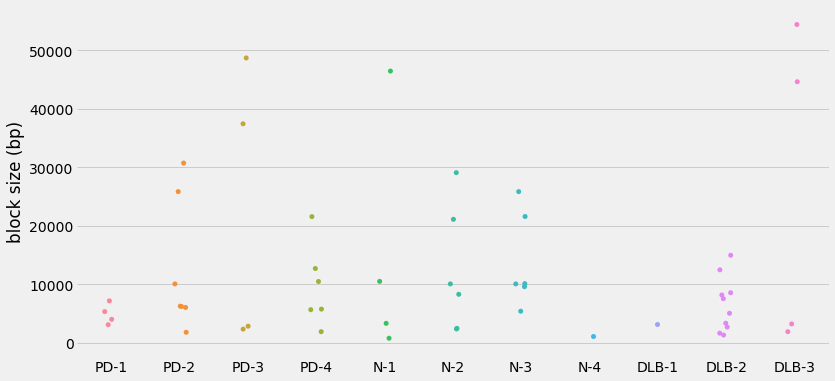


**(b)**


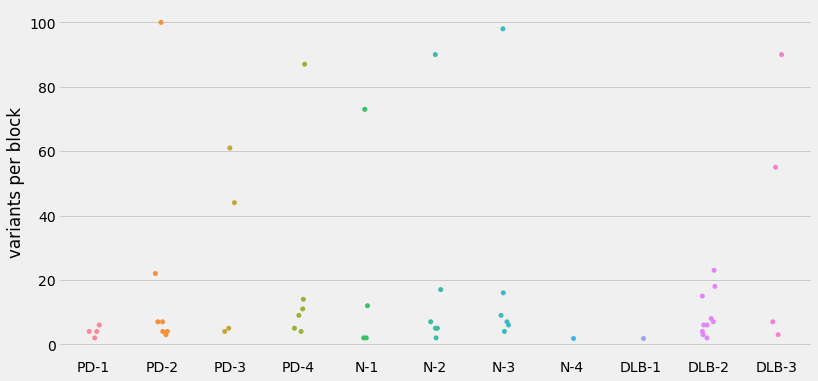


**Supplementary Figure 4. PacBio cDNA (Iso-Seq) bioinformatics workflow** as implemented in the IsoSeq3 application in PacBio’s SMRT Analysis v6.0 software. Full-Length reads are identified based on the presence of cDNA primers, barcodes, and polyA tail, then clustered at the isoform-level and polished to obtain high-quality consensus sequences. The high-quality sequences are then mapped back to the genome for downstream analysis.


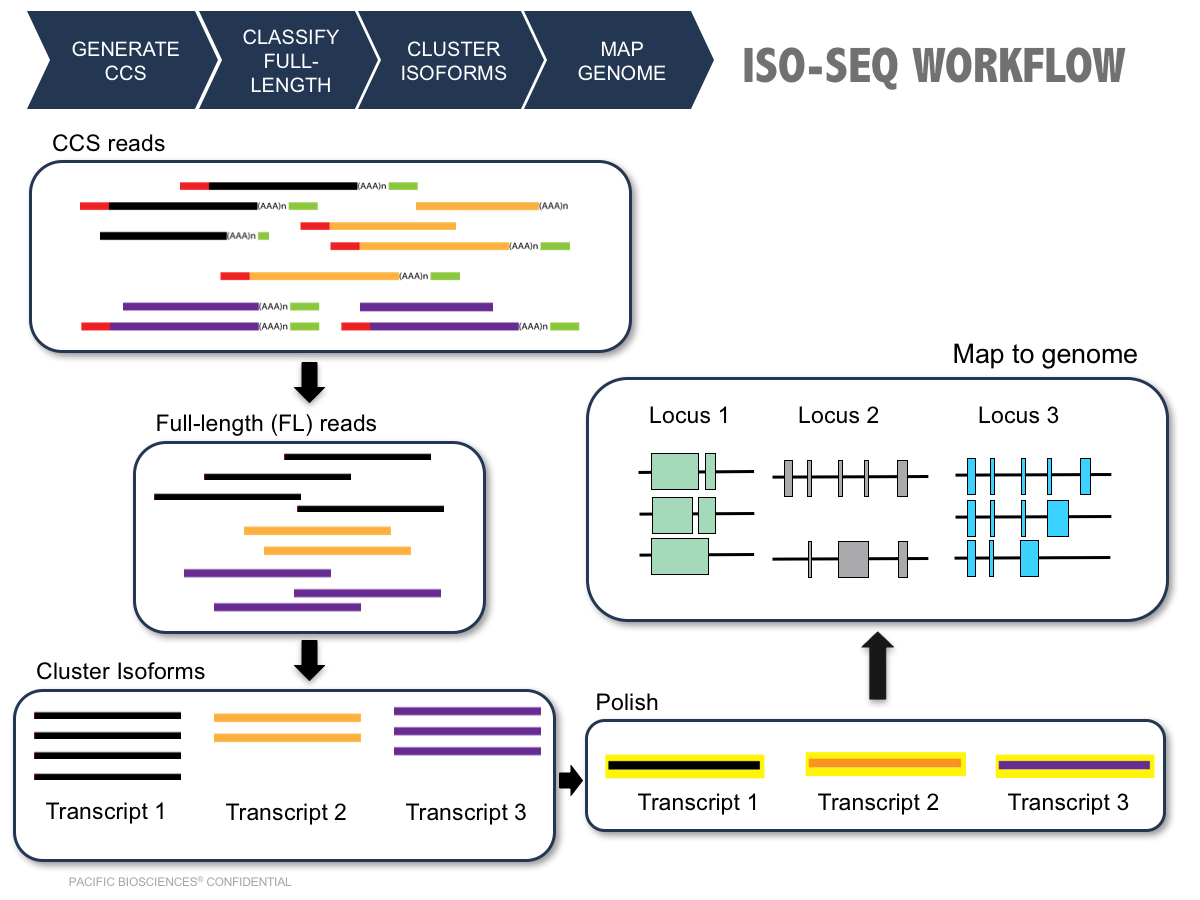


**Supplementary Figure 5. Open Reading Frame prediction and alignment** for the final isoforms. Identical predicted ORFs are deduplicated. *canonical*: isoforms containing all 6 exons with only 5’ and 3’ UTR variability; *alt5*: alternative isoforms using a novel start site in intron 4; *alt3*: alternative isoforms using novel end sites in intron 4; *skip3*: alternative isoforms that skip exon 3; *skip5*: alternative isoforms that skip exon 5; *rare*: isoforms with rare alternative splice sites in exon 1, 2, 4 (colored green and blue in main Figure 3).


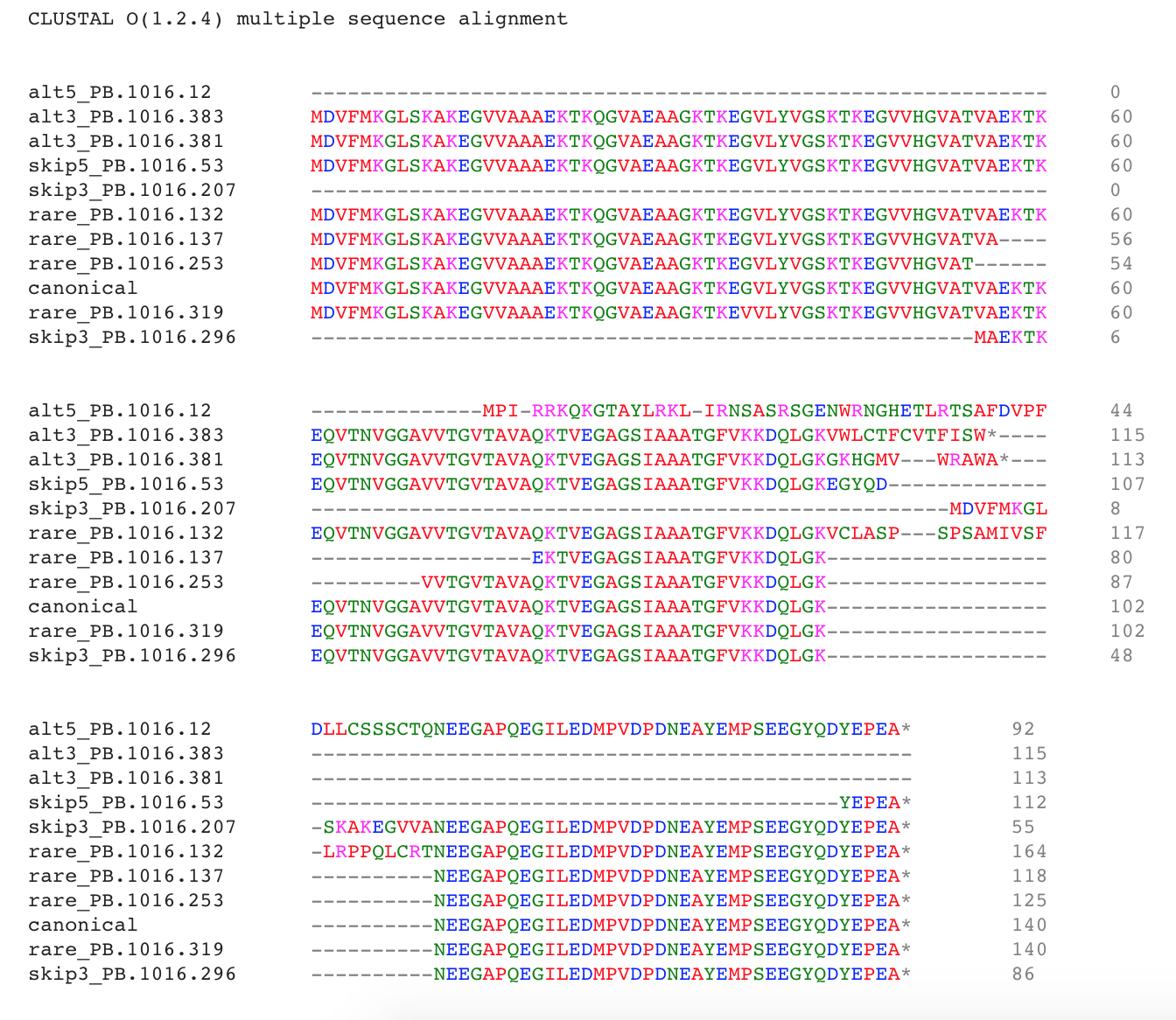


**Supplementary Table 1.** **Demographic information of the 12 samples.** PD=Parkinson’s Disease. N=Normal. DLB=Dementia with Lewy Body. PMI=post mortem interval. RIN= RNA integrity number. The barcode index is used to identify the sample after multiplexing.

| Sample | Barcode | Age at Death | Gender | PMI | RIN |
| --- | --- | --- | --- | --- | --- |
| PD-1 | 1 | 83 | M | 2 | 6.2 |
| PD-2 | 2 | 78 | F | 3.5 | 5.5 |
| PD-3 | 3 | 85 | M | 2.66 | 8.2 |
| PD-4 | 4 | 88 | M | 2 | 8.1 |
| N-1 | 5 | 86 | M | 2.5 | 8.1 |
| N-2 | 6 | 72 | F | 30 | 6.5 |
| N-3 | 7 | 87 | M | 18.5 | 8.3 |
| N-4 | 8 | 78 | M | 9.83 | 8.2 |
| DLB-1 | 9 | 74 | M | 17.85 | 6.7 |
| DLB-2 | 10 | 89 | F | 43 | 5.3 |
| DLB-3 | 11 | 79 | M | 10.93 | 8.4 |
| DLB-4 | 12 | 71 | M | 9.25 | 6.4 |

**Supplementary Table 2. Variants Detected in Targeted gDNA Data.** For each type of SNPs in different regions, the number of novel/known (dbSNP) variants are shown. A total of 8 SNPs, 13 indels and 10 SSRs variants were called.

|  | SNPs  (novel/known) | Indels  (novel/known) | SSR variants |
| --- | --- | --- | --- |
| exon 1 (5’ UTR) | 0/5 | 0/0 | 0 |
| intron 1 | 0/0 | 0/0 | 0 |
| exon 2 (5’ UTR) | 0/0 | 0/0 | 0 |
| exon 2 (coding) | 0/0 | 0/0 | 0 |
| intron 2 | 1/46 | 0/2 | 0 |
| exon 3 | 0/0 | 0/0 | 0 |
| intron 3 | 1/59 | 0/6 | 1 (2bp repeat unit),  2 (3bp repeat unit),  2 (4bp repeat unit) |
| exon 4 | 0/0 | 0/0 | 0 |
| intron 4 | 6/160 | 13/13 | 1 (2bp repeat unit);  2 (3bp repeat unit);  1 (4bp repeat unit) |
| exon 5 | 0/0 | 0/0 | 0 |
| intron 5 | 0/1 | 0/0 | 0 |
| exon 6 (coding) | 0/0 | 0/0 | 0 |
| exon 6 (3’UTR) | 0/3 | 0/1 | 1 (2bp repeat) |

**Supplementary Table 3.** Number of demultiplexed full-length (FL) reads for each patient sample.

| Sample | Barcode | FL Reads |
| --- | --- | --- |
| PD-1 | 1 | 6,298 |
| PD-2 | 2 | 1,391 |
| PD-3 | 3 | 7,277 |
| PD-4 | 4 | 11,909 |
| N-1 | 5 | 22,739 |
| N-2 | 6 | 17,880 |
| N-3 | 7 | 19,770 |
| N-4 | 8 | 8,579 |
| DLB-1 | 9 | 6,323 |
| DLB-2 | 10 | 5,937 |
| DLB-3 | 11 | 18,545 |
| DLB-4 | 12 | 3,343 |
| TOTAL |  | 129,991 |

**Supplementary Table 4.** **SNCA isoform abundance**. The abundance for each isoform is the fraction of on-target, full-length reads associated with that isoform.

| **GROUP** | **PBID** | **PD-1** | **PD-2** | **PD-3** | **PD-4** | **N-1** | **N-2** | **N-3** | **N-4** | **DLB-1** | **DLB-2** | **DLB-3** | **DLB-4** |
| --- | --- | --- | --- | --- | --- | --- | --- | --- | --- | --- | --- | --- | --- |
| **AllExons** | **PB.1016.10** | 0.1% | 0.0% | 0.1% | 0.2% | 0.1% | 0.0% | 0.1% | 0.0% | 0.0% | 0.0% | 0.1% | 0.0% |
| **AllExons** | **PB.1016.47** | 1.5% | 1.5% | 1.2% | 1.3% | 1.3% | 1.1% | 1.3% | 0.4% | 1.1% | 0.9% | 1.8% | 2.2% |
| **AllExons** | **PB.1016.44** | 7.6% | 0.0% | 7.2% | 5.0% | 8.2% | 6.4% | 7.8% | 3.4% | 3.8% | 4.7% | 9.6% | 3.2% |
| **AllExons** | **PB.1016.45** | 0.7% | 0.0% | 1.1% | 0.7% | 1.0% | 1.7% | 1.0% | 1.0% | 0.4% | 0.6% | 1.9% | 0.5% |
| **AllExons** | **PB.1016.52** | 0.0% | 0.0% | 0.1% | 0.0% | 0.1% | 0.1% | 0.1% | 0.0% | 0.0% | 0.0% | 0.2% | 0.0% |
| **AllExons** | **PB.1016.108** | 0.0% | 0.0% | 0.0% | 0.0% | 0.0% | 0.1% | 0.0% | 0.0% | 0.1% | 0.0% | 0.0% | 0.0% |
| **AllExons** | **PB.1016.118** | 0.4% | 0.0% | 0.2% | 0.2% | 0.2% | 0.2% | 0.2% | 0.2% | 0.0% | 0.1% | 0.3% | 0.3% |
| **AllExons** | **PB.1016.139** | 0.1% | 0.0% | 0.0% | 0.0% | 0.1% | 0.1% | 0.1% | 0.0% | 0.0% | 0.2% | 0.1% | 0.3% |
| **AllExons** | **PB.1016.138** | 0.0% | 0.0% | 0.0% | 0.1% | 0.0% | 0.1% | 0.1% | 0.0% | 0.0% | 0.0% | 0.0% | 0.3% |
| **AllExons** | **PB.1016.137** | 0.0% | 0.0% | 0.0% | 0.1% | 0.0% | 0.0% | 0.0% | 0.1% | 0.1% | 0.0% | 0.1% | 0.0% |
| **AllExons** | **PB.1016.136** | 0.1% | 0.0% | 0.2% | 0.2% | 0.1% | 0.1% | 0.1% | 0.2% | 0.4% | 0.2% | 0.2% | 0.2% |
| **AllExons** | **PB.1016.142** | 7.8% | 0.0% | 5.9% | 9.3% | 6.4% | 8.5% | 8.5% | 5.1% | 9.8% | 9.1% | 7.3% | 12.3% |
| **AllExons** | **PB.1016.169** | 0.0% | 0.0% | 0.1% | 0.1% | 0.1% | 0.1% | 0.0% | 0.1% | 0.0% | 0.0% | 0.0% | 0.0% |
| **AllExons** | **PB.1016.131** | 54.6% | 55.9% | 56.4% | 59.0% | 63.7% | 55.0% | 59.7% | 62.4% | 57.4% | 57.5% | 52.6% | 53.9% |
| **AllExons** | **PB.1016.119** | 5.0% | 11.8% | 4.4% | 3.2% | 2.9% | 3.2% | 2.6% | 2.9% | 3.3% | 2.1% | 3.4% | 3.0% |
| **AllExons** | **PB.1016.123** | 7.2% | 8.8% | 9.3% | 8.9% | 7.2% | 12.6% | 8.7% | 11.6% | 9.3% | 8.5% | 9.2% | 8.5% |
| **AllExons** | **PB.1016.253** | 0.2% | 0.0% | 0.1% | 0.2% | 0.1% | 0.1% | 0.1% | 0.2% | 0.2% | 0.2% | 0.5% | 0.2% |
| **AllExons** | **PB.1016.252** | 0.1% | 0.0% | 0.0% | 0.1% | 0.0% | 0.1% | 0.1% | 0.2% | 0.0% | 0.1% | 0.0% | 0.0% |
| **AllExons** | **PB.1016.177** | 0.4% | 0.0% | 0.2% | 0.3% | 0.3% | 0.4% | 0.3% | 0.4% | 0.1% | 0.3% | 0.2% | 0.6% |
| **AllExons** | **PB.1016.170** | 2.1% | 5.9% | 2.5% | 1.4% | 1.6% | 2.1% | 1.3% | 1.4% | 3.8% | 3.8% | 2.3% | 3.8% |
| **AllExons** | **PB.1016.275** | 2.1% | 1.5% | 2.6% | 2.0% | 1.7% | 3.5% | 2.0% | 3.7% | 2.1% | 2.9% | 2.1% | 2.5% |
| **AllExons** | **PB.1016.132** | 0.3% | 0.0% | 0.1% | 0.3% | 0.2% | 0.0% | 0.0% | 0.0% | 0.0% | 0.2% | 0.2% | 0.0% |
| **AllExons** | **PB.1016.273** | 1.8% | 0.0% | 1.3% | 0.8% | 0.8% | 0.8% | 0.6% | 0.8% | 0.5% | 0.6% | 0.8% | 1.1% |
| **AllExons** | **PB.1016.319** | 0.3% | 0.0% | 0.1% | 0.2% | 0.1% | 0.0% | 0.1% | 0.2% | 0.2% | 0.2% | 0.1% | 0.0% |
| **AllExons** | **PB.1016.322** | 3.2% | 11.8% | 2.2% | 1.9% | 1.6% | 0.8% | 1.2% | 2.2% | 2.3% | 1.6% | 1.1% | 1.9% |
| **AllExons** | **PB.1016.324** | 0.0% | 0.0% | 0.1% | 0.0% | 0.0% | 0.0% | 0.0% | 0.0% | 0.2% | 0.0% | 0.1% | 0.0% |
| **AllExons** | **PB.1016.321** | 0.2% | 0.0% | 0.3% | 0.2% | 0.2% | 0.2% | 0.2% | 0.3% | 0.5% | 0.3% | 0.2% | 0.5% |
| **AllExons** | **PB.1016.334** | 0.3% | 0.0% | 0.1% | 0.2% | 0.2% | 0.2% | 0.2% | 0.1% | 0.3% | 0.3% | 0.2% | 0.9% |
| **Skip3** | **PB.1016.296** | 0.0% | 0.0% | 0.0% | 0.0% | 0.0% | 0.0% | 0.0% | 0.1% | 0.0% | 0.1% | 0.1% | 0.0% |
| **Skip3** | **PB.1016.207** | 0.0% | 0.0% | 0.0% | 0.1% | 0.0% | 0.1% | 0.0% | 0.2% | 0.0% | 0.0% | 0.2% | 0.0% |
| **Skip5** | **PB.1016.53** | 0.4% | 1.5% | 0.1% | 0.1% | 0.1% | 0.0% | 0.1% | 0.0% | 0.0% | 0.1% | 0.3% | 0.3% |
| **Skip5** | **PB.1016.128** | 0.4% | 0.0% | 0.5% | 0.2% | 0.1% | 0.2% | 0.1% | 0.2% | 0.8% | 0.3% | 0.4% | 0.5% |
| **Skip5** | **PB.1016.143** | 0.2% | 0.0% | 0.1% | 0.0% | 0.0% | 0.1% | 0.0% | 0.1% | 0.1% | 0.1% | 0.1% | 0.2% |
| **Skip5** | **PB.1016.199** | 0.1% | 0.0% | 0.2% | 0.2% | 0.1% | 0.1% | 0.1% | 0.2% | 0.2% | 0.1% | 0.0% | 0.2% |
| **Skip5** | **PB.1016.161** | 1.5% | 0.0% | 0.9% | 0.5% | 0.5% | 0.5% | 0.7% | 0.8% | 1.0% | 0.8% | 0.7% | 0.8% |
| **Alt5** | **PB.1016.12** | 0.0% | 0.0% | 0.0% | 0.0% | 0.0% | 0.0% | 0.1% | 0.0% | 0.0% | 0.0% | 0.1% | 0.0% |
| **Alt5** | **PB.1016.41** | 0.3% | 0.0% | 0.3% | 0.5% | 0.1% | 0.1% | 0.4% | 0.0% | 0.1% | 0.3% | 1.0% | 0.0% |
| **Alt5** | **PB.1016.127** | 0.8% | 0.0% | 1.3% | 2.2% | 0.6% | 0.6% | 1.3% | 0.1% | 0.8% | 2.5% | 2.1% | 0.8% |
| **Alt3** | **PB.1016.381** | 0.0% | 0.0% | 0.0% | 0.0% | 0.0% | 0.0% | 0.0% | 0.3% | 0.0% | 0.1% | 0.0% | 0.2% |
| **Alt3** | **PB.1016.383** | 0.1% | 1.5% | 0.0% | 0.0% | 0.0% | 0.1% | 0.0% | 0.2% | 0.0% | 0.2% | 0.1% | 0.2% |
| **Alt3** | **PB.1016.384** | 0.5% | 0.0% | 0.9% | 0.2% | 0.4% | 0.4% | 0.5% | 1.1% | 0.9% | 0.7% | 0.4% | 0.8% |

**Supplementary Methods**

**Isoform Bioinformatics Analysis**

Bioinformatics analysis was done by running the Iso-Seq 3 application in the PacBio SMRT Analysis v6.0 to obtain high-quality, full-length transcript sequences, followed by downstream analysis. The workflow consists of the following steps:

**Identify full-length reads.** Full-length reads were determined as CCS reads that contained both the 5’ and 3’ primer and the polyA tail preceding the 3’ primer. The 5’ primer consists of the Clontech SMARTer cDNA primer with an ATGGG overhang. The 3’ primer consists of a 16-bp PacBio barcode that is patient-specific followed by the Clontech SMARTer cDNA primer.

The 5’ primer is identical for all patient samples: AAGCAGTGGTATCAACGCAGAGTACATGGG

The 3’ primer for the 12 patient samples are below:

PD-1: CGCACTCTGATATGTGGTACTCTGCGTTGATACCACTGCTT

PD-2: CTCACAGTCTGTGTGTGTACTCTGCGTTGATACCACTGCTT

PD-3: CTCTCACGAGATGTGTGTACTCTGCGTTGATACCACTGCTT

PD-4: CGCGCGTGTGTGCGTGGTACTCTGCGTTGATACCACTGCT

N-1: ACGCGAGAGTCGAGTGGTACTCTGCGTTGATACCACTGCTT

N-2: ACAGCTGATATATATGGTACTCTGCGTTGATACCACTGCTT

N-3: CACATAGAGATACAGAGTACTCTGCGTTGATACCACTGCTT

N-4: CGCAGCGCTCGACTGTGTACTCTGCGTTGATACCACTGCTT

DLB-1: TCTGTCTCGCGTGTGTGTACTCTGCGTTGATACCACTGCTT

DLB-2: CTCTGAGATAGCGCGTGTACTCTGCGTTGATACCACTGCTT

DLB-3: ATAGATATACGTATAGGTACTCTGCGTTGATACCACTGCTT

DLB-4: ACACGCGATCTAGTGTGTACTCTGCGTTGATACCACTGCTT

**Isoform-level clustering analysis to obtain high-quality transcript sequences.** To increase detection of rare isoforms, the de-multiplexed FL reads were pooled to perform isoform-level clustering analysis (Gordon et al. 2015). After clustering, consensus sequences are called using the Arrow algorithm and only polished sequences with predicted consensus accuracy ≥99% were considered high-quality (HQ) and retained for the next step.

**Mapping to hg19 and filtering for on-target isoforms.** The HQ transcript sequences were mapped to hg19 using minimap2 (version 2.11-r797) using parameters ` -ax splice -t 30 -uf --secondary=no -C5`. We then filtered transcripts mapped to the SNCA gene region on chromosome 4 on the minus strand (chr4: 90637000-90770000) with ≥99% coverage and ≥99% identity.

**Removing potential PCR artifacts and obtaining the final set of on-target SNCA isoforms**. We applied further filtering criteria to remove potential genomic contamination and rare PCR artifacts. Each final SNCA isoform must be: (a) supported by a total of 20 FL reads; (b) does not have ≥ 80% genomic ‘A’s in the 3’ downstream 20-bp window; (c) all splice junctions are canonical; and (d) no junctions are predicted to be template switching artifacts. We used a custom script to filter for 20 FL reads and applied SQANTI2 (<https://github.com/Magdoll/SQANTI2/>), a modified version of the SQANTI tool (Tardaguila et al. 2017) to filter out criteria (b)-(d). We obtained a final set of 41 SNCA isoforms.

**De-multiplex final isoforms by sample**. We calculated the relative abundance of each the final isoforms in each sample by extracting the fraction of FL reads supporting each isoform from each sample.

**Open Reading Frame prediction**. We error corrected any remaining sequence errors by using the hg19 genome bases, then predicted open reading frames (ORFs) using the software ANGEL v2.4 (<https://github.com/PacificBiosciences/ANGEL)>. The predicted amino acid sequences were aligned using Clustal Omega (<https://www.ebi.ac.uk/Tools/msa/clustalo/)>.

**SNP calling using Iso-Seq data**. All full-length reads from all 12 samples were aligned to the hg19 genome using minimap2 (v2.11-r797) to create a pileup. Then, at each position with at least 10 base coverage, a Fisher exact test with Bonferroni correction is applied with a p-value cutoff of 0.01. Only substitution SNPs are called. Then, sample-specific haplotype information is obtained by looking at the number of FL reads associated with each allele. The phasing code, named IsoPhase, could be found at the GitHub Cupcake repository (<https://github.com/Magdoll/cDNA_Cupcake>).

**REFERENCES**

Gordon, Sean P, Elizabeth Tseng, Asaf Salamov, Jiwei Zhang, Xiandong Meng, Zhiying Zhao, Dongwan Kang, et al. 2015. “Widespread Polycistronic Transcripts in Fungi Revealed by Single-Molecule mRNA Sequencing.” Edited by Deyou Zheng. *PLoS ONE* 10 (7). Public Library of Science: e0132628. doi:10.1371/journal.pone.0132628.

Tardaguila, Manuel, Lorena de la Fuente, Cristina Marti, Cecile Pereira, Francisco Jose Pardo-Palacios, Hector del Risco, Marc Ferrell, et al. 2017. “SQANTI: Extensive Characterization of Long Read Transcript Sequences for Quality Control in Full-Length Transcriptome Identification and Quantification,” August, 1–31. doi:10.1101/118083.
